# Takayasu Arteritis with Severe Renal Artery Stenosis and an Aberrant Renal Artery in a Child: A Case Report and Discussion

**DOI:** 10.31138/mjr.230727.bt

**Published:** 2023-07-27

**Authors:** Manas Ranjan Behera, Manoranjan Mohapatra, Sushrith Kumar Yadav

**Affiliations:** 1 Department of Paediatrics, Kalinga Institute of Medical Sciences (KIMS), KIIT Deemed University, Patia, Bhubaneswar, Odisha, India,; 2 Department of Radiodiagnosis, Kalinga Institute of Medical Sciences (KIMS), KIIT Deemed University, Patia, Bhubaneswar, Odisha, India

**Keywords:** Takayasu arteritis, vasculitis, renal artery stenosis, haematuria, renal artery abnormalities

## Abstract

Takayasu arteritis (TA) is a chronic, idiopathic large vessel vasculitis mainly affecting the aorta and its major branches. It is one of the common causes of reno-vascular hypertension in Indian children. We report a ten-year-old boy who presented with hypertensive encephalopathy, proteinuria, and haematuria without any renal dysfunction. He was initially diagnosed to be a case of acute post streptococcal glomerulonephritis, but detailed clinical examination and haemato-radiological investigations revealed Takayasu arteritis, type V (P+). He had unilateral severe renal artery stenosis along with a small kidney and an aberrant renal artery on left side. He is found to have resistant hypertension, unresponsive to multiple anti-hypertensive drugs, and had a fatal outcome. This case illustrates renal involvement in TA and the significance of four-limb blood pressure measurement in any non-obese child with hypertension. Furthermore, the possible role of aberrant renal artery in the pathogenesis of resistant hypertension is discussed.

## INTRODUCTION

Takayasu arteritis (TA) is an idiopathic granulomatous large vessel vasculitis predominantly affecting the aorta, its major branches, and less frequently, the pulmonary arteries.^1^ It occurs more commonly in females in the second and third decade of life but has been reported in infants also.^2^ The incidence of TA in adults is estimated to be 1/1,000,000/year in Europe and 2.6/1,000,000/year in North America.^3,4^ Though the exact incidence in children is not known, it is found to be more prevalent in certain geographical regions like Central and South America, Africa, India, and the Far East.^5^ It is the most common cause of renovascular hypertension in India.^6^ Renal artery stenosis can be unilateral or bilateral. Unilateral affection can present with hypertension, and bilateral involvement may result in hypertension with or without renal failure. Haematuria can be a rare presentation of TA. Most children with the renovascular disease will need interventional or surgical treatment. Here we describe a ten-year- old boy, who presented with haematuria and hypertensive encephalopathy; magnetic resonance angiography (MRA) showed extensive vascular involvement suggestive of Takayasu arteritis, with an aberrant renal artery on the left side and a small kidney.

## CASE DESCRIPTION

A ten-year-old boy was admitted with a history of oliguria and macroscopic haematuria for the last two days. He had two attacks of generalised tonic-clonic seizure on the day of admission. He had headaches and vomiting for the last two days. There was no history of fever, trauma, seizure, or any urinary symptoms before. There was no family history of hypertension, renal disease, or seizure disorder.

On clinical examination, the child was drowsy and irritable. He was afebrile, pale-looking, without any icterus or oedema. He weighed 25 kg (less than 50th percentile) and his height was 135 cm (50th percentile). Heart rate was 110/min, regular with good volume and all the peripheral pulsations were well palpable. Blood pressure, recorded in left upper limb was 138/90 mm of Hg (>95^th^ percentile). There was mild hepatomegaly without any splenomegaly or lymphadenopathy. Other systemic examination findings were unremarkable. Bilateral fundi examination showed no evidence of papilledema. In view of the presence of haematuria, oliguria, hypertension, and seizure, a diagnosis of acute post-streptococcal glomerulonephritis (APGN) with hypertensive encephalopathy was made and investigated accordingly. He had haemoglobin 10.1 gm/dl with a microcytic, hypochromic picture, total leucocyte count 9,100/cmm (N 88, L 10, M 2), and total platelet count 5.28 lac/MM3. Serum urea (17 mg/dl), creatinine (0.52 mg/dl) and electrolytes (Na 132 mmol/l, K 3.7 mmol/l, C1 mg/dl) were normal. Urine examination showed mild proteinuria and microscopic haematuria (15–20 RBC/HPF) without any RBC casts. Serum antistreptolysin-O (ASO) titre was positive (400; Normal up to 320 Todd units), but complement C3 (183 mg/dl), C4 (32 mg/dl) levels were normal. ESR was 76 mm in 1st hour and CRP was high (25.68 mg/L). Brain CT was found to be normal.

**Figure 1.**
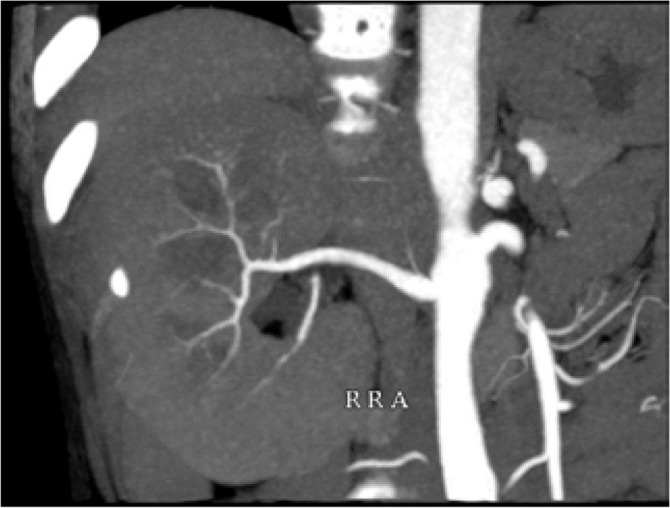
MR angiogram showing left side main renal artery (LRA) stenosis with small size kidney (6.2 x 3.2 cm).

**Figure 2.**
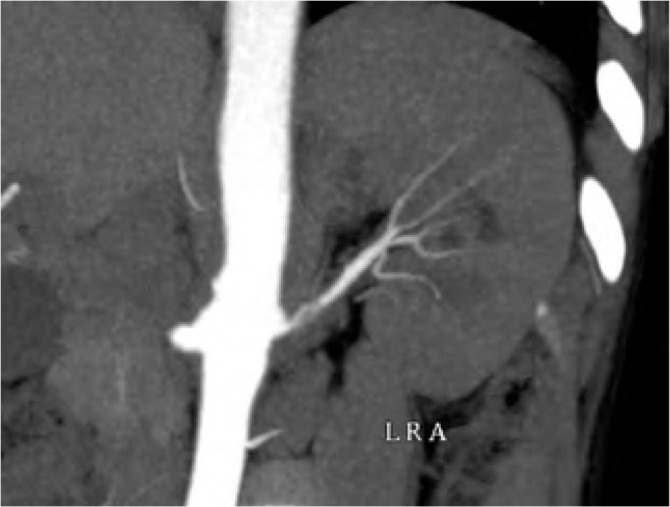
MR angiogram showing right side normal calibre renal artery (RRA) with normal size kidney (9.2 x 3.8 cm).

**Figure 3.**
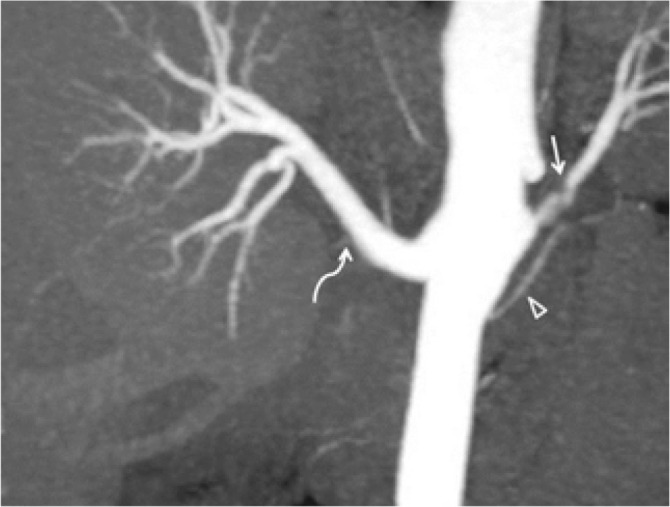
Maximum intensity projection coronal image from CT Angiography depicts stenosis in the main renal artery (arrow) and patent aberrant renal artery (arrowhead) on left along with normal calibre right renal artery (curved arrow).

**Figure 4.**
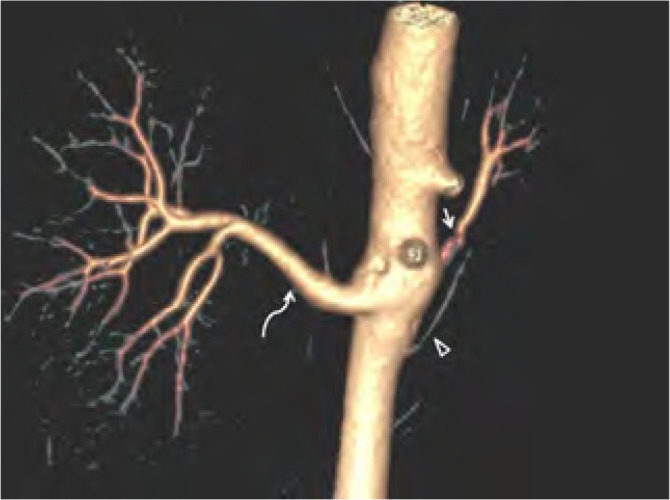
Volume rendered coronal image from CT Angiography depicts stenosis in the main renal artery (arrow), patent aberrant renal artery (arrowhead) on left with normal calibre right renal artery (curved arrow).

In view of normal renal function tests, C3, and C4 levels, the provisional diagnosis of AGN seemed unlikely. He was re-examined and investigated thoroughly. His blood pressure in right and left brachial artery was 138/90 mm and 150/100 mm Hg respectively. Blood pressure in right and left lower limbs were found to be 164/112 mm and 150/108 mm Hg. There was no carotid or renal bruit and no carotidynia. His serum was negative for antinuclear antibody, antinuclear cytoplasmic antibody, and rheumatoid factor. Serology for HBsAg, HCV, and HIV was negative. The chest radiograph was normal. No coronary artery abnormalities were found out on 2 D echocardiography. The tuberculin test was positive (16 mm). Ultrasonography of the abdomen showed hepatomegaly (15.7 cm) and smaller size left kidney (6.2 x 3.2 cm). Bilateral renal arteries doppler study showed significant left main renal artery stenosis. Magnetic resonance angiogram (MRA) of systemic circulation showed mural thickening of the left common carotid artery at its origin and proximal part of descending thoracic aorta without any evidence of luminal stenosis. The abdominal aorta was affected (enhanced wall thickness) to a greater length (6.6 cm) involving the origins of the superior mesenteric artery, celiac trunk, and bilateral renal arteries. The severe luminal narrowing was seen in the left renal artery at its origin and proximal part (**Figure 1**). The part distal to the narrowing also showed a mild reduction in calibre. The left kidney was smaller in size but showed normal shape and density with reduced contrast enhancement as compared to right (**Figure 2**). An aberrant renal artery was seen on the left side supplying the lower pole of the left kidney arising from the aorta approximately 11.8 mm below the origin of left main renal artery (MRA) which was well visualised on renal CT angiography (**Figure 3**, **Figure 4**). Multiple enlarged para-aortic, peripancreatic and axillary node were seen, largest measuring 1.0 x 1.8 cm in size. MRA of pulmonary circulation showed mural thickening (2.1mm thickness, length 3.1 cm) of the descending branch of the left pulmonary artery, other parts being normal. Cerebral vasculature was normal. DTPA scan showed extremely poor functioning left kidney contributing less than 10% to the GFR.

In view of the above findings, a diagnosis of Takayasu aortoarteritis type V (P+) with renovascular hypertension and hypertensive encephalopathy was made. He was initially treated with IV fluids, nifedipine, and phenytoin on admission. After two days he became fully conscious and seizure-free. Prednisolone was started at 2 mg/kg upon confirmation of the diagnosis by MRA. As the blood pressure was remaining persistently high during his stay of almost one and half months, labetalol, clonidine, hydralazine, and prazosin were added in a stepwise manner. He was also started on Mycophenolate mofetil (900mg/d). Ultimately, he was on multiple antihypertensive drugs namely, nifedipine (2.4 mg/kg/d), clonidine (17 mcg/kg/d), labetalol (6 mg/kg/day), hydralazine (0.75 mg/kg/d) and prazosin (0.2mg/kg/d). Multiple consultations were done with rheumatologist, urologist, cardiothoracic surgeon, and cardiologist. A decision of left side nephrectomy was taken as the child did not attain clinical remission with the pharmacotherapy, which was essential before any surgical procedure such as percutaneous transluminal angioplasty, stenting, or bypass surgery, and the presence of an almost non-functioning left kidney with uncontrolled hypertension. Parents were counselled and explained to in detail about the procedure and prognosis of the condition. However, they were unwilling for any kind of surgical intervention and wanted to consult some other hospital. Hence, he was advised to continue with the anti-inflammatory and antihypertensive drugs and discharged. After two weeks the boy was readmitted to the hospital in a moribund condition. He died after four days due to congestive cardiac failure and multi-organ dysfunction.

## DISCUSSION

The diagnosis of TA can be a challenge, especially in the initial phases. There is no diagnostic serologic test and symptoms are generally constitutional, including malaise, fever, fatigue, and arthralgia. Early initiation of immune suppressive treatment is crucial to control active inflammation and minimise arterial injury. Our patient presented with haematuria, oliguria, and hypertension mimicking acute post-streptococcal glomerulonephritis. Though ASO titre was found to be positive, other features like C3, C4, serum urea, and creatinine levels were within normal range. The discrepancy in four limb blood pressure, which was missed earlier, and finding of renal artery stenosis on doppler ultrasonography gave clue to the diagnosis. Renal Doppler ultrasonography should be the first line of investigation in children with hypertension. Though conventional angiography is the gold standard for the diagnosis of TA, it does not give information on vessel wall thickening, oedema, and inflammation, which is picked up accurately by MRA.^7^ Currently, it is the imaging tool of choice in the early diagnosis of TA. Our patient had severe left renal artery stenosis along with mural thickening involving left common carotid, descending thoracic aorta, abdominal aorta, and pulmonary artery without any luminal stenosis as evidenced by MR angiography. Hence, he was diagnosed to be a case of Takayasu arteritis type V, P (+) as per the ACR criteria as well as the revised EULAR/PReS criteria.^8^

Persistent haematuria in TA can be due to ischemic injury or glomerular involvement rarely. Mesangial proliferation is the predominant histological feature reported in glomerulonephritis associated with TA without renal artery stenosis.^9^ Our patient had haematuria most probably due to glomerular ischemia caused by renal artery stenosis. Supernumerary renal arteries are variants in normal vascular anatomy which are often thought to be harmless. The accessory renal artery usually arises from the aorta above or below the main renal artery (MRA) and enters the hilum, whereas aberrant renal artery arises from the aorta and pierces the upper or lower pole supplying a part of the kidney.^10^ They are longer, narrower, more prone to develop stenosis, and may have had some role in the pathogenesis of reno-vascular hypertension.^11^ Though the incidence is reported to be 25–30%, we could not find any case of TA along with the accessory or aberrant renal artery in the literature. This may contribute to resistant hypertension through renin dependent hyperaldosteronism.^12,13^ They are narrower, longer, and prone to stenosis, causing under perfusion of part of the kidney. Though controversial, still the role of such vascular anomaly in severe and difficult to manage hypertension in children cannot be denied.^13,14^

Clinical features of TA include nonspecific findings in early-stage (pre-pulseless phase) like a low-grade fever, night sweats, malaise, weight loss, arthralgia, and fatigue. The later stage (pulseless phase) is characterised by inflammatory and obliterative changes in the arteries manifested by diminished or absent pulse, hypertension (renal artery stenosis), renal bruit, abdominal angina, retinopathy, aortic regurgitations, syncope, and seizure; however, there may be an overlap between the two stages.^15^ The basic pathology is a persistent inflammation of all the vessel layers resulting in stenosis, occlusion, dilatation, or aneurysm formation. The prevalence of renal artery involvement in patients of TA varies from 11.5% to 62%, which is higher in Asian population as compared to North America, Northern Europe and Africa.^16^ Childhood TA is the leading cause of paediatric hypertension secondary to renal artery stenosis or mid-aorta stenosis in Asian countries.^17^ Surgical options available are percutaneous transluminal renal arterial dilatation (PTRA), renal artery stenting, and surgical bypass. Though these surgical procedures have a short-term benefit in children with TA, a high failure rate in the long term is a matter of concern.^18^ In our case, the stenosis in the left renal artery involved a longer segment, and angioplasty or stent replacement was not possible. From a technical point of view, the surgical bypass procedure of the stenosis was also not feasible. The child had resistant hypertension, despite being on five antihypertensive drugs. As the renal scintigraphy showed very minimal function in the left kidney along with an aberrant renal artery, nephrectomy was planned on that side.

High dose glucocorticoids (Prednisolone 1–2 mg/kg) are the cornerstone of the management of TA. Other drugs used are methotrexate, cyclophosphamide, azathioprine, mycophenolate mofetil, and infliximab. Our patient was started with prednisolone and later on mycophenolate mofetil was added, but was unresponsive to the drugs. Childhood TA is associated with mortality as high as 35%.^15^ Causes of death include arterial dissection, aortic rupture, uncontrollable hypertension, cardiomyopathy, myocardial infarction, renal failure, and infection. Our case succumbed to congestive heart failure and multi-organ dysfunction.

## CONCLUSION

Takayasu arteritis is a poorly understood disease with a grave prognosis having variable presentations, which throws many challenges for treating physicians. Atypical presentation and an incidental finding of an anatomical variation make this case unique. Awareness, a high index of suspicion of the disease, and detailed clinical examination, particularly four-limb (or at least two upper limb) blood pressure measurement is crucial to early diagnosis in a hypertensive child, who may present with haematuria. Doppler ultrasonography should be the first line of investigation and MR angiography should be the imaging modality of choice to know the disease extension, activity, and follow up.

## ABBREVIATIONS:

ACR: American College of Rheumatology
CRP: C-reactive Protein
CT: Computerised Tomography
DTPA: Diethylenetriamine Pentaacetate
ESR: Erythrocytic sedimentation rate
EULAR: European Alliance of Associations for Rheumatology
GFR: Glomerular filtration rate
IV: Intravenous
PReS: Paediatric Rheumatology European Society

## DECLARATION

Our case report titled “Takayasu arteritis with severe renal artery stenosis and an aberrant renal artery in a child: A case report and discussion” has not been published in whole or in part elsewhere and not currently being considered for publication in another journal.
